# The Cost-Effectiveness of Congenital Adrenal Hyperplasia Newborn Screening in Brazil: A Comparison Between Screened and Unscreened Cohorts

**DOI:** 10.3389/fped.2021.659492

**Published:** 2021-05-24

**Authors:** Mirela Costa de Miranda, Luciana Bertocco de Paiva Haddad, Evelinda Trindade, Alex Cassenote, Giselle Y. Hayashi, Durval Damiani, Fernanda Cavalieri Costa, Guiomar Madureira, Berenice Bilharinho de Mendonca, Tania A. S. S. Bachega

**Affiliations:** ^1^Unidade de Adrenal da Disciplina de Endocrinologia, Laboratório de Hormônios e Genética Molecular/LIM42, Hospital das Clínicas da Faculdade de Medicina da Universidade de São Paulo, São Paulo, Brazil; ^2^Divisão de Transplantes Hepático, Departamento de Gastroenterologia, Hospital das Clínicas da Faculdade de Medicina da Universidade de São Paulo, São Paulo, Brazil; ^3^São Paulo State Health Technology Assessment Network, São Paulo State Department of Health, São Paulo, Brazil; ^4^Laboratório do Serviço de Referência em Triagem Neonatal, Instituto Jô Clemente, São Paulo, Brazil; ^5^Unidade de Endocrinologia Pediátrica Do Instituto da Criança, Hospital das Clínicas da Faculdade de Medicina da Universidade de São Paulo, São Paulo, Brazil

**Keywords:** cost-effectiveness, newborn screening, economic evaluation, 21-hydroxilase deficiency, congenital adrenal hyperplasia

## Abstract

**Background:** Newborn screening for congenital adrenal hyperplasia (CAH-NBS) is not yet a worldwide consensus, in part due to inconclusive evidence regarding cost-effectiveness because the analysis requires an understanding of the short- and long-term costs of care associated with delayed diagnosis.

**Objective:** The present study aimed to conduct a cost-effectiveness analysis (CEA) to compare the costs associated with CAH-NBS and clinical diagnosis.

**Methods:** A decision model comparing the two strategies was tested by sensitivity analysis. The cost analysis perspective was the public health system. Unscreened patients' data were extracted from medical records of Hospital das Clinicas, Saõ Paulo, and screened data were extracted from the NBS Referral Center of São Paulo. The population comprised 195 classical patients with CAH, clinically diagnosed and confirmed by hormonal/*CYP21A2* analysis, and 378,790 newborns screened during 2017. Adverse outcomes related to late diagnosis were measured in both cohorts, and the incremental cost-effectiveness ratio (ICER) was calculated. We hypothesized that CAH-NBS would be cost-effective.

**Results:** Twenty-five screened infants were confirmed with CAH (incidence: 1:15,135). The mortality rate was estimated to be 11% in unscreened infants, and no deaths were reported in the screened cohort. Comparing the unscreened and screened cohorts, mean serum sodium levels were 121.2 mEq/L (118.3–124.1) and 131.8 mEq/L (129.3–134.5), mean ages at diagnosis were 38.8 and 17 days, and hospitalization occurred in 76% and 58% of the salt-wasting patients with the in the two cohorts, respectively. The NBS incremental cost was US$ 771,185.82 per death averted, which yielded an ICER of US$ 25,535.95 per discounted life-year saved.

**Conclusions:** CAH-NBS is important in preventing CAH mortality/morbidity, can reduce costs associated with adverse outcomes, and appears cost-effective.

## Introduction

Congenital adrenal hyperplasia (CAH) due to 21-hydroxylase is a common autosomal recessive disease that results in impaired cortisol and/or aldosterone synthesis and excessive adrenal androgen secretion (1, 2). The worldwide and Brazilian incidence ranges from 1:10,000 to 1:18,000 (3, 4). The disease presents in two clinical forms: the classical form with severe enzyme deficiency and prenatal onset of virilization, and the non-classical form with mild enzyme deficiency and postnatal onset. The classical form is divided into the salt-wasting (SW) and simple virilizing (SV) forms. Female newborns with both clinical forms present with virilized external genitalia at birth; in the SW form, infants of both sexes are also at risk of life-threatening adrenal crisis in the absence of early treatment (1). Benefits of precocious diagnosis of SW cases through newborn screening (NBS) include reduced mortality and reduced length of hospitalization, while in the SV form, benefits include the correction of sex assignment errors in virilized females and prevention of early advanced bone maturation (5).

Although false-negative cases have been reported (6, 7), CAH-NBS has demonstrated good efficacy in detecting patients with classical CAH, and its implementation is a recommendation by the Endocrine Society (5). However, the high frequency of false-positive (FP) results remains a major issue, especially in preterm newborns. The positive predicted value (PPV) has been described to be ~1.6–2.3% in screened Brazilian cohorts (8–11). A recent pilot study conducted in Saõ Paulo found a PPV of 5.6 and 14.1% upon using the 99.8th percentile in samples collected until and after 72 h of life, respectively (3). Neonates with positive screening results should undergo additional tests to confirm the diagnosis, and this process increases screening costs (12, 13).

CAH-NBS implementation is not yet a worldwide consensus, which, in part, reflects the inconclusive data regarding the cost-effectiveness of early CAH diagnosis through NBS (12). The lack of consensus has been highlighted in the recent CAH clinical guidelines (5). Long-term experience with CAH-NBS has been described; however, only a few studies have compared the screened and unscreened populations (14–16). Two cost-effectiveness analyses (CEAs) concerning CAH-NBS have been conducted in the United States, and in assuming varying mortality rates, they reached differing conclusions: one concluded that CAH-NBS was cost-effective, while the other did not (17, 18). A recent CEA performed in Canada reached a conclusion that favored NBS based on avoided morbidity and hospital costs, even though no deaths were apparently averted (19). None of these analyses considered the long-term adverse outcomes secondary to late diagnosis and all were performed in high-income countries, in which CAH can be precociously diagnosed clinically.

Clinical suspicion of CAH in the absence of acute crisis is commonly due to either genital atypia identification in females or a family history of CAH. Thus, most males can be clinically diagnosed only during a neonatal SW crisis in the SW form or due to hyperandrogenic manifestations during early infancy in the SV form. The delayed SW form may incur longer hospitalizations, and some patients may die without correct diagnosis (5, 20–22). In patients with the SV form, delayed diagnosis leads to precocious pubarche and/or precocious pseudo-puberty, culminating in a short final height and hyperandrogenic manifestations in females (23, 24). It is known that genital atypia at birth can become unnoticed, and highly virilized females could also be misassigned at birth as males (22, 25).

The major objective of our study was to assess the cost-effectiveness of CAH-NBS in terms of prevention of early childhood death and comorbidities with a set of evidence-based assumptions. This study aimed to offer a comprehensive cost-effectiveness evaluation of CAH-NBS implementation by using a decision-tree framework that allowed variation in the model parameters, considering the costs related to CAH screening compared to those related to the treatment of short- and long-term adverse outcomes of late CAH diagnosis.

## Methods

A CEA was conducted to compare the strategies of CAH-NBS and clinical recognition using data from two CAH cohorts diagnosed by NBS and clinically. Frequencies of adverse outcomes were evaluated in both cohorts and used to construct the decision model. The study design followed the recommendations for economic health evaluation (26).

The study was approved by the Ethics in Research Committees of the entities involved in providing data (online register number 14558). The data were retrospectively extracted from the medical records of unscreened patients from the main tertiary Hospital of Saõ Paulo State and from the medical records of screened patients from the Neonatal Screening Referral Center of Saõ Paulo.

The CEA was conducted from the perspective of the Brazilian Public Health System (SUS), which includes healthcare costs but not families' costs. It used the values of reimbursements paid by the Brazilian Government for each health procedure, obtained directly from the Management System of the Brazilian Public Health System Table (*SIGTAP)* through the Brazilian Ministry of Health online platform (27). The monetary unit was United States dollars (US$), which was converted from the Brazilian currency (R$) using the 2016 exchange rate of US$ 1.00 = R$ 3.26 (28). The chosen time horizon was from diagnosis to 19 years, a period in which most outcomes occur.

The unscreened population comprised 195 patients (141 F) diagnosed by clinical suspicion and confirmed by hormonal and molecular analyses. Mortality rate, dehydration parameters, and hospitalization length were measured in SW cases. The number of deaths due to the absence of diagnosis was calculated by applying the calculated mortality rate to the proportion of patients with the SW form who would have not been clinically recognized.

A fixed amount is paid by the SUS forward hospitalizations, regardless of the length of stay; however, intensive care unit (ICU) care is paid on a per-day basis. Therefore, the costs related to dehydration were related to the number of ward hospitalizations, as well as the number and duration of ICU hospitalizations. In SV cases, the analysis evaluated the frequency of patients with the 46,XX karyotype raised as males and of those undergoing gonadotropin-releasing hormone analog (GnRha) and growth hormone (GH) therapies for precocious pseudo-puberty in both sexes (29). The costs related to therapies for males with the 46,XX karyotype were due to genital surgery and androgen replacement from puberty to 19 years, and costs related to GnRha and GH use were calculated according to the number of patients treated, mean dose, and treatment duration.

In the screened population, the analysis used data from the CAH-NBS program of the state of Saõ Paulo in 2017, in which 378,790 newborns were screened. Eighty-two percent of the samples were collected in hospitals, 11% in public health units that provided primary care for the population, 6.3% in private laboratories, and 0.6% in private clinics. Data on birth date, weight, sex, date of sampling, neonatal 17-hydroxyprogesterone (17-OHP) levels, and serum confirmatory tests were recorded. NBS was performed by heel prick capillary blood collection from newborns between the 2nd and 7th days of life. The blood was deposited on a S&S 903 filter paper, and dried samples were transported to the referral laboratory for 17-OHP analysis using time-resolved, solid-phase fluoroimmunoassay 17-OHP neonatal kit (B015-112–AutoDELFIA Neonatal 17-α-OH-progesterone kit; Perkin Elmer Life and Analytical Sciences, Wallac Oy, Turku, Finland). The intra-assay and inter-assay coefficients were <8% for the filter paper assay. The cutoffs for 17-OHP levels on the filter paper were established according to the birth weight and age at sample collection using the 99.8th percentile (3). Newborns with increased 17-OHP levels that were less than twice the reference value (low-risk) were submitted for a new sample collection on a filter paper; samples that had 17-OHP levels higher than twice the 99.8th percentile (high risk) were submitted for serum confirmatory tests, and those patients were directly referred to a specialist consultation. Serum confirmatory tests included measurements of 17-OHP, androstenedione, and cortisol levels by liquid chromatography-mass spectrometry (LC-MS /MS); of testosterone by eletroquimioluminescence; and of sodium and potassium by selective ion electrode.

Screening costs included 17-OHP measurements on the filter paper, in addition to confirmatory tests and medical follow-ups for all screened positive cases. It was calculated as the cost of measuring the 17-OHP level from the same filter paper collected for the other screening tests performed by the national NBS program. Costs by the category of screened newborns are shown in Table 1. In newborns with confirmed CAH diagnosis costs, our decision model included the frequency and length of hospitalization due to dehydration. It was assumed that under the screening strategy, adverse long-term outcomes would not occur as all patients would be diagnosed early. Additionally, no deaths due to SW crises were reported in the NBS cohort.

**Table 1:** Costs for different categories after CAH screening until the age of 1 year old.

**Categories**	**Risk at screening**	**Costs description**	**Cost per child (US$)**
Non-affected by CAH		A	2.45
Non-affected by CAH retested		2A	4.9
False positive	High	A + B	24.91
	Low	2A + B	27.36
CAH affected	High	A + B	24.91
	Low	2A + B	27.36

*A—17OHP on filter paper = $2.45*.

*B—Confirmatory laboratory (serum 17OHP $3.13, cortisol $3.02, androstenedione $3.54, testosterone $3.20, sodium $0.57, and potassium $0.57) + medical consultation ($8.43) = $22.46*.

**Figure 1:**
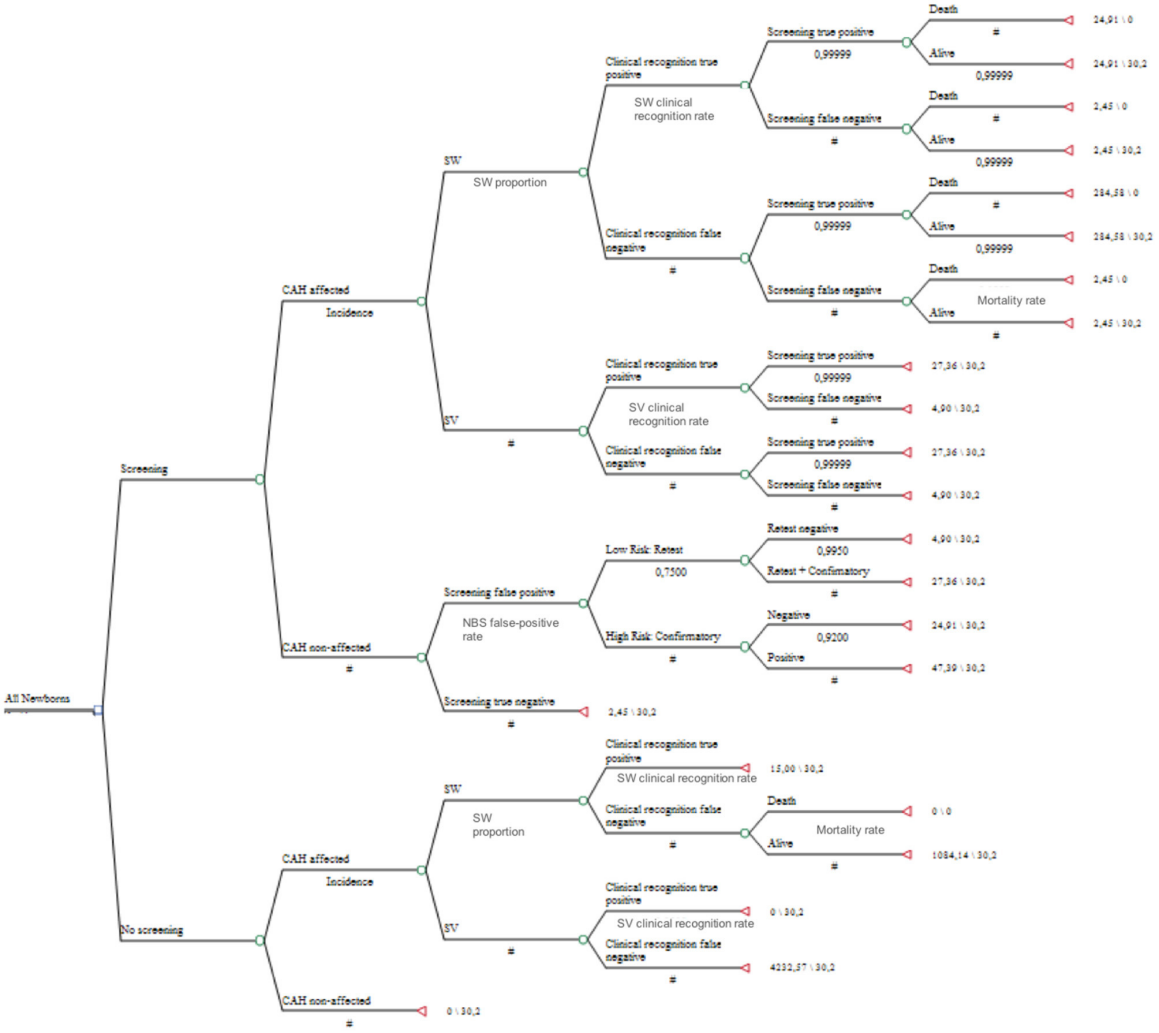
The design of the decision tree analysis.

Figure 1 shows the decision tree. It calculated the incremental cost-effectiveness ratio (ICER) as the ratio of the incremental cost of CAH-NBS to the discounted life-years saved (LY). Following a model also used by Yoo and Grosse (17), our analyses assumed that each newborn's death avoided would save 30.2 LYs, applying a 3% discount rate to 76 years of life expectancy at birth, as per the 2017 Brazilian life table (30). The equation used was as follows: ICER (US$/LY) = (costs of NBS arm—costs of non-screened arm) / (number of deaths averted × 30.2 LYs). The analyses were conducted using the decision analysis software program TreeAge Pro Health Care, and the results were confirmed by Excel analysis.

Table 2 lists all parameters used in the CEA, including point estimates for the base case. The CAH incidence of 1:12,250 is an average of the incidence estimates we found in this study (1:15,135) and different ones described previously by other Brazilian centers (4, 11). We assumed a 75% proportion of SW cases, as it has been widely reported in the literature and also in a Brazilian NBS study (5, 8). Clinical recognition within the NBS cohort was the probability of a case being detected before the presentation of symptoms due to genital atypia at birth or family history before the reporting of NBS results. This probability was based on the proportion of patients with the SW form diagnosed before dehydration and those with the SV form diagnosed in the first 30 days of life in our unscreened cohort (20); the average between these proportions and the one previously published by Yoo and Grosse was used in the base case analysis (17). The mortality rate considered in the base case analysis was also an average of the one calculated in our unscreened cohort and another published previously (17). The decision model was applied to a hypothetical cohort using the total number of live births in Brazil in 2017, surveyed from the Brazilian Ministry of Health database (31) (see Table 3).

**Table 2:** Parameters and its point estimate assumption in modeling the base, best- and worst-case scenarios in decision analysis for CAH-NBS.

**Parameters**	**Point of estimate**			**References**
	**Base case**	**Best case**	**Worst case**	
2017 Brazilians life expectation and LY saved after 3% discount	76–30.2	76–30.2	76–30.2	(30)
CAH incidence	1:12,250	1:10,000	1:15,000	(11), Actual study
% SW	75%	75%	75%	(5, 8)
Screening false positive rate	0.2%	0.1%	0.5%	(17), Actual study
High risk false positive	25%	25%	25%	Actual study
False positive confirmatory	8%	8%	8%	Actual study
Low risk false positive	75%	75%	75%	Actual study
Retest false positive	0.5%	0.5%	0.5%	Actual study
**SW form**				
% clinical recognition	36%	16%	55%	(17)
SW mortality without screening	8%	11%	4.2%	(17, 20)
% hospitalization even with screening	58%	58%	58%	Actual study
% ICU hospitalization even with screening (among hospitalized patients)	30%	30%	30%	Actual study
Screened length of ICU (days)	9	9	9	Actual study
% hospitalization unscreened	91%	91%	91%	(20)
% ICU of unscreened, among hospitalized patients	36%	36%	36%	
Unscreened length of ICU (days)	23	23	23	
**SV form**				
% clinical recognition	28%	11%	45%	(17, 20)
% use of GH	14%	14%	14%	(20)
Length of GH treatment (years)	3.2	3.2	3.2	
Mean GH dose (ui)	5.9	5.9	5.9	
% Females submitted to Masculinization process	15%	15%	15%	
% use of GnRha	28%	28%	28%	
Length of GnRha treatment (years)	3.8	3.8	3.8	
**Monetary reimbursements values**	**US$**			
Nursery hospitalization	70.8			(23)
ICU diary	155.9			
Neonatal 17OHP screening test (A)	2.45			
Confirmatory laboratory + medial consultation (B)	22.46			
GH ampole 12 ui	33.97			
GnRH ampole 11.75 mg	273.75			
Masculinization process	1,906.19			
Laboratory diagnosis (17OHP, andro, testo, Na, K, renin)	15.05			

**Table 3:** CEA for CAH-NBS in Brazil during the year of 2017.

**Decision model on a hypothetic population**	**Base case**	**Best case**	**Worst case**
Yearly live-births (*N*)	2,923,535	2,923,535	2,923,535
Yearly CAH affected newborns (*N*)	239	292	195
SW newborns (*N*)	179	219	146
SV newborns (*N*)	60	73	49
**SCREENING STRATEGY**			
**SW form**			
Clinical recognition (*N*)	64	35	80
Costs (A + B) (US$)	1,594.24	871.85	1,992.80
Deaths (*N*)	0	0	0
Hospitalizations (*N*)	0	0	0
Total costs (US$)	1,594.24	871.85	1,992.80
No clinical recognition (*N*)	115	184	66
Costs (A + B) (US$)	2,864.65	4,583.44	1,644.06
Deaths (*N*)	0	0	0
Hospitalizations (*N*)	67	107	38
ICU (*N*)	20	32	11
ICU length (days)	9	9	9
Hospitalizations costs (US$)	32,805.60	52,474.80	18,124.50
Total costs (US$)	35,670.25	57,058.24	19,768.56
**SV form**			
Costs (2A + B) (US$)	1,641.60	1,997.28	1,340.64
Deaths	0	0	0
Late adverse outcomes (N)	0	0	0
Total costs (US$)	1,641.60	1,997.28	1,340.64
Non-affected (*N*)	2,923,296	2,923,243	2,923,340
True negatives (*N*)	2,917,449	2,920,320	2,908,723
Costs (A) (US$)	7,147,750.05	7,154,784.00	7,126,371.35
False positives (*N*)	5,847	2,923	14,617
High risk (*N*)	1,462	731	3,654
Confirmatory true negative (*N*)	1,345	673	3,362
Costs (A + B) (US$)	33,503.95	16,764.43	83,747.42
Confirmatory false positive (*N*)	117	58	292
Costs (A + 2B) (US$)	5,542.29	2,747.46	13,832.04
Low risk (*N*)	4,385	2,192	10,963
Retest true negative (*N*)	4,363	2,181	10,908
Costs (2A) (US$)	21,378.70	10,686.90	53,449.20
Retest false positive (*N*)	22	11	55
Costs (2A + B) (US$)	601.92	300.96	1,504.80
Total cost of screening strategy (US$)	7,247,683.00	7,245,211.12	7,302,006.81
Deaths (*N*)	0	0	0
**NON-SCREENING STRATEGY**			
**SW form**			
Clinical recognition (*N*)	64	35	80
Deaths (*N*)	0	0	0
Hospitalizations (*N*)	0	0	0
Diagnostic lab (US$)	963.20	526.75	1,204.00
Total costs (US$)	963.20	526.75	1,204.00
No clinical recognition (*N*)	115	184	66
Deaths (*N*)	9	20	3
Hospitalizations (*N*)	96	149	57
ICU (*N*)	35	54	21
ICU length (days)	23	23	23
Hospitalization costs (US$)	132,296.30	204,177.00	79,335.30
Dehydration without hospitalization (cost of lab exams) (US$)	150.05	225.75	90.30
Total costs (US$)	132,446.80	204,402.75	79,425.60
**SV form**			
Clinical recognition (*N*)	17	8	22
Costs with lab diagnostic (US$)	255.85	120.40	331.10
No clinical recognition (*N*)	43	65	27
Number of 46XX reared as males (*N*)	3	5	2
Masculinization process (US$)	5,718.57	9,530.95	3,812.38
Number of patients with GH treatment (*N*)	6	9	4
Use of GH cost (US$)	117,047.03	175,570.55	78,031.35
Number of patients with GnRh treatment (*N*)	12	18	8
Use of GnRha cost	49,932.00	74,898.00	33,288.00
Costs with lab diagnostic (US$)	647.15	978.25	406.35
Total costs (US$)	174,160.23	260,977.75	115,538.08
Total cost of non-screening strategy (US$)	307,010.60	466,027.65	196,498.78
Deaths (*N*)	9	20	3
Incremental cost of screening (US$)	6,940,672.40	6,779,183.47	7,105,508.03
Deaths averted (*N*)	9	20	3
Incremental cost per death averted (US$)	771,185.82	338,959.17	2,368,502.68
Incremental cost per life years saved (US$)	25,535.95	11,223.81	78,427.24

*SW, salt-wasting form; SV, simple virilizing form; LY, years of life saved; N, number; US$, American dollar*.

Considering the inherent uncertainty in the values of various assumptions, the robustness of the model was tested by applying a one-way sensitivity analysis to examine how the ICER was influenced by the range of each parameter. The sensitivity analysis involved four variables, which are listed in Table 4. In addition, analyses of the “best-case” and “worst-case” scenarios selected the most and least favorable values for each parameter, respectively. The ranges were extracted from the published literature.

**Table 4:** CEA sensitivity analysis.

**One-way sensitivity analysis of base-case analysis**	**Range**	**ICER (US$/LY)**
CAH incidence	1:10,000	20,972.42
	1:15,000	33,337.73
Clinical recognition SW/SV	16/11%	19,189.90
	55/45%	38,959.02
SW mortality without NBS	11%	17,870.08
	4.2%	46,387.70
False positive rate	0.1%	25,423.64
	0.5%	25,872.61
Cohort of date birth from 2000		36,630.47

*CEA, cost-effectiveness analysis; SW, salt-wasting; SV, simple virilizing; LY, years of life saved; NBS, newborn screening*.

### Statistical Analysis

Data are expressed as mean and 95% confidence intervals (CIs). The Student's *t-*test, chi-square test, and Fisher exact test were used. Statistical significance was set at *P* < 0.05. Openepi software was used, which is available at https://www.openepi.com/.

## Results

### Unscreened CAH Cohort

The cohort consisted of 195 patients (90 SV and 105 SW cases), and SW cases comprised 54% of the patients. Considering that CAH clinical awareness could vary over time, the results were stratified according to the year of birth: Group 1 until 1989, Group 2 from 1990 to 1999, and Group 3 after 1999. The SW frequency was 43% in Group 1 (94 patients), 62% in Group 2 (68 patients), and 70% in Group 3 (33 patients). The mortality rate was assumed to be 11%, obtained by subtracting the mean SW frequency of the most recent cohorts of Groups 2 and 3 (64%) from that found in a Brazilian screened cohort (75%) (8).

In the sensitivity analysis, data from Group 3 were used to analyze whether the improvement in CAH awareness impacted CEA. Group 3 comprised 33 patients (22 F) born between 2000 and 2014; 23 had the SW (14 F) form and 10 (9 F) had the SV form. One patient with the 46, XX karyotype who had the SV form was raised as male (11%). Dehydration occurred in 20 of the 23 patients with the SW form (87%), hospitalization occurred in 18 (90%), and 61% were admitted to the ICU for a mean duration of 20 days (95% CI, 6.2–33.8). GH treatment was used in 40% of the patients for a mean duration of 3.9 years (95% CI, 2.7–5.99) and a mean dose of 5.09 UI/day (95% CI, 3.9–15.9). Treatment with GnRHa (11.25 mg/3 months) was used in 30% of the patients for a mean duration of 5.1 years (95% CI, 3.0–7.2).

SW crises occurred in 84% of the patients (100% of men and 77% of women). Therefore, the probability of clinical recognition before the presentation of symptoms in the SW form was calculated to be 16%. The mean serum sodium (Na) level was 121.2 mEq/L (95% CI, 118.3–124.1), and the mean age at diagnosis was 38.8 days (95% CI, 31.1–46.4). Hospitalization occurred in 91% of the dehydrated patients (76% of all patients with the SW form) for a mean duration of 38 days (95% CI, 30.3–47.4); 36% were in the ICU for a mean duration of 23 days (95% CI, 13.2–32.1). Table 5 shows the main clinical differences between the screened and unscreened patients with the SW form.

**Table 5:** Clinical differences between unscreened and screened patients.

**Variables**	**Unscreened**	**Screened**	***p value***
Age at SW diagnosis	38.8 days	17.3 days	<0.0001
Mean Na level at diagnosis	121.2 mEq/L	131.8 mEq/L	<0.0001
% hospitalization (of all SW patients)	76%	58%	0.058
% ICU	29%	36%	0.041
Length of ICU	22.9 days	9 days	0.042

*SW, salt-wasting form; SV, simple virilizing form; Na, sodium; ICU, intensive care unit*.

Among the 90 patients with the SV form, 67 (73.6%) were women. Atypical genitalia was identified at birth in 28 patients (44%). Only 11% of all patients with the SV form were diagnosed with CAH during the neonatal period, which was the assumed probability of clinical recognition. Ten out of 67 females (15%) were assigned at birth as males and were raised as males and submitted to the masculinization process after extensive psychological evaluation. The mean difference between bone age and chronological age at diagnosis was 5.2 years in both sexes (95% CI, 4.4–5.9). Twenty-five patients (28%) were treated with GnRHa (11.25 mg/3 months) for a mean period of 3.8 years (95% CI, 3.08–4.5). GH therapy was used in 13 patients (14%) for a mean period of 3.2 years (95% CI, 2.4–4.0), and the mean dose was 5.9 UI/day (95% CI, 4.9–6.8).

### Newborn Screening for CAH

In 2017, there were 611,803 live births in the state of Saõ Paulo, and ~63% of the newborns were screened by the Jo Clemente Institute, which performs NBS in the city of Saõ Paulo and some other neighboring cities, not the entire state. The total coverage rate for NBS in Saõ Paulo State is ~88%. A total of 378,379 newborns were screened in 2017. Among them, 25 were affected by the classical forms of CAH, with an incidence of 1:15,135 live births. Recall was performed in 4,677 newborns (1.23%), wherein 3,810 newborns had very low birth weight (<1,500 g) and 435 newborns had neonatal transfusion. The CEA considered only the recall of altered neonatal 17-OHP results. The mean duration of neonatal sample collection was 5.25 days of life (95% CI, 4.87–5.82) and of that between sample collection and the NBS result was 5.16 days (95% CI, 4.93–5.40). A total of 843 unaffected newborns presented with altered 17-OHP levels and a 0.22% false-positive rate on the first-tier screen. Death due to prematurity and neonatal complications occurred in 321 newborns, of which 22 had altered 17-OHP levels; however, information from their hospitalizations and laboratory examinations ruled out a diagnosis of CAH. Among the false-positive cases, 75% were considered low risk and a retest on filter paper was performed; 99.5% were dismissed after a negative retest result and the other 0.5% were dismissed after serum confirmatory tests and medical consultation. Among the 25% who were considered high risk, 92% were dismissed after a negative serum confirmatory test, and 8% remained inconclusive and needed a second confirmatory test.

Of the 25 CAH cases, 10 were male and 15 were female. Despite NBS, 12 patients presented with dehydration, 11 were hospitalized, and 30% were in the ICU with a mean length of stay of 9 days (95% CI, 1.8–16.2). The mean sodium level of the patients who were dehydrated was 131.8 mEq/L (95% CI, 129.3–134.5). The mean sodium level at diagnosis of all the patients was 133.7 mEq/L (95% CI, 131.5–135.8). The mean duration between the filter paper sample collection and first medical consultation was 12 days (95% CI, 7.8–16.1) and mean age at first medical consultation was 17.2 days (95% CI, 11.3–23.3). Among the females, 8 out of 15 were hospitalized due to dehydration. As the patients were not followed up at the same center and molecular diagnosis was not performed, we could not precisely estimate the proportion of patients with the SW form. If we consider this expected proportion to be 75% (8), 19 patients would be salt-wasters; hence, the hospitalization rate would be 58% (11/19).

### Cost-Effectiveness Analysis

In a hypothetical cohort of 2,923,535 live births per year, using the Brazilian live-birth rate of 2017 (31), with a CAH incidence of 1:12,250, we would have had 239 newborns with CAH and 179 (75%) cases with the SW form. Under the NBS strategy, the total cost would be US$ 7,247,683.00 (US$ 30,368.75 per patient diagnosed with CAH). The total cost in this unscreened scenario would be US$ 307,010.60, with 9 neonatal deaths. Therefore, the incremental cost of screening was US$6,940,672.40, and the effectiveness was 9 deaths averted, i.e., US$ 771,185.82 per death averted. After dividing that number by 30.2, discounted LY yields an ICER of US$ 25,535.95/LY saved in the base case analysis. The CEA analysis results are presented in Table 3.

Variations in the ICERs according to each variable are shown in Table 4. With a set of assumptions favoring CAH screening, the best-case analysis yielded an ICER of US$ 11,223.81/LY. The worst-case analysis showed an ICER of US$ 78,427.24/LY. The SW-form mortality and clinical recognition variables had the greatest influence on ICER. The 4.2% to 11% mortality rate variation led to an ICER of US$ 46,387.70 to US$ 17,870.08, respectively, whereas the assumption of 55% clinical recognition of the SW form and 45% of the SV form led to an ICER of US$ 38,959.02/LY, while the assumptions of 16% and 11%, respectively, led to an ICER of US$ 19,189.90/LY. Additionally, the ICER calculated using the data of only the most recent cohort birth from 2000 to 2014 was US$ 36,630.47/LY.

## Discussion

Recommendations for NBS are primarily based on evidence of the importance and certainty of net benefits (32). While NBS saves lives and improves the quality of lives of affected children and their families, an analysis of its economic impact is important to avoid overloading the public health system (33).

In accordance with published data on CAH-NBS, this analysis of CAH-NBS showed that although half of the screened patients still had to be hospitalized due to dehydration, the severity and length of hospitalization were significantly lower than those of the unscreened cohort (15, 19, 34, 35). A possible reason for this might have been the delay in obtaining the NBS results. The mean age at diagnosis in the screened cohort was 17.2 days, a period in which the SW crisis may have already occurred. Other NBS programs also face similar problems (19, 34, 36). Clearly, this issue presents implications for the cost-effectiveness of NBS, and its improvement must be targeted by NBS programs (37). Respecting the recommended age at sample collection of 3–5 days and improving the subsequent flow in obtaining the NBS results would allow patients to be earlier diagnosed and therefore decrease the hospitalization rate and costs.

Studies on CAH-NBS have focused on the benefits of preventing SW-related crises and have not considered adverse outcomes related to the late diagnosis of the SV form, which also has economic impacts (20, 38). In the absence of NBS, newborns with the SV form are generally unrecognized at birth and can develop, in early infancy, precocious pseudo-puberty with increased growth velocity and rapid bone age maturation (39). This explains the frequent use of expensive therapies, such as GnRha and GH, in our cohort, which could have been avoided with an earlier diagnosis. The mean age at diagnosis of 5.7 years was sufficient to warrant advanced bone maturation in almost 50% of the patients (20). Although GnRha and GH are not part of the standard treatment regimen for CAH, they are considered in the CAH Guidelines of the Endocrine Society as alternative treatments for very low predicted final height and precocious puberty (5). In our experience of following these patients that were diagnosed late and not screened, we observed that these outcomes had a great impact on the patients' and families' quality of lives and also increased the costs of treatment; therefore, we included these outcomes in our CEA. To the best of our knowledge, this was the first CEA for CAH-NBS to consider these outcomes.

Traditionally, interventions in the United States are assumed to be cost-effective if the ICER is up to US$ 50,000/LY (40). The World Health Organization considers a strategy cost-effective if the ICER costs <3 times the gross domestic product (41), which in Brazil would be US$ 29,294.63 in 2017 (42). Thus, our study showed that CAH-NBS is likely to be cost-effective in the Brazilian context. The worst-case analysis suggested that in a set of assumptions not favorable to NBS, such as low mortality, high standards of clinical awareness, and lower CAH incidence, CAH-NBS would not be considered cost-effective. However, we consider this scenario less likely to occur, especially in a low-to-middle-income country context.

We observed that the two variables that most affected the ICER were mortality rate and the ability to clinically recognize the disease during the pre-symptomatic phase. Interestingly, these two variables differ most between high- and low-to-middle-income countries, demonstrating the importance of carrying out economic studies using the population characteristics of each region. CAH-NBS is reportedly not performed in the United Kingdom because of a lack of evidence for increased mortality in the absence of NBS (43). In contrast, other developed countries that performed CAH-NBS assumed that it was effective in reducing morbidity and mortality (19, 44, 45). The assumed risk of death for patients with the SW form in the absence of NBS ranges widely across Europe and North America, from 0 to 9% (12, 19, 46). However, even though these evaluations were performed among populations with high standards of CAH clinical awareness, it might be possible that there was some underestimation in relation to undiagnosed cases (5, 22).

The observations of higher CAH incidence after CAH-NBS implementation and of unbalanced female/male and SW/SV ratios in some unscreened populations supported our findings of higher CAH mortality due to unrecognized SW-related crises (5, 22, 47, 48). Although we previously estimated that the mortality rate may reach 26%, we decided to be more conservative and used the lowest mortality rate calculated previously (11%), and also considered the mortality rate used in another CEA carried out in the United States, a high-income country (4.2%) (17, 20). It's worth noting that mortality among unscreened CAH infants is almost tenfold higher than the one estimated for the general Brazilian infant population in 2017 (49). Additionally, this analysis did not include indirect costs related to parents' work absenteeism and the direct and indirect costs of mental impairment due to severe neonatal hyponatremia (20), although its prevention is recognized as a potential benefit of early diagnosis (5, 50, 51) because doing so could overestimate the ICER for NBS.

Clinical recognition of CAH was estimated based on the frequency of case detection before the presentation of symptoms due to genital atypia at birth or family history. In an ideal situation, all female newborns would be diagnosed at birth due to genitalia atypia; however, we showed that only 21% of females with the SW form were prevented from dehydration due to early recognition. These data explained why our clinical recognition probabilities were lower than those assumed in previous studies (17). Other studies examining screened CAH populations in developed countries have also reported that up to 38% of females were diagnosed only by NBS, highlighting the importance of screening for CAH in both sexes (15, 19, 36, 52).

Our study had several limitations. Data regarding the parameters of our decision model were derived from a study performed in a specific state of Brazil. In addition, we used the Brazilian public health system's economic data to calculate costs, and large variations in costs may occur among different countries. These reimbursement amounts might have been underestimated, especially in the unscreened cohort, which could have underestimated the ICER. This, in addition to some methodological variations, might explain why our ICER results were not comparable to those of previous studies (17–19). To deal with the uncertainty in the variables, we conducted sensitivity analyses and used data published in other studies on CAH.

Another limitation was that, it was not possible to ensure that no cases were missed in our 1-year analysis of CAH-NBS. False-negative results have been reported in other screened populations (6), which could be related to early sample collection. In our cohort, heel prick collection was performed after 48 h of life, and the cut-off values were in accordance with birth weight, which guarantees good sensitivity and specificity for screening results (3, 11, 53). The incidence observed in our screened cohort of São Paulo in 2017 was slightly lower than that previously reported (4, 11). Although no missed case was reported, the female-to-male ratio suggests otherwise. It might have been possible that some males with the SV form were missed, possibly due to the antenatal use of glucocorticoids, which is a frequent practice in obstetrician centers in São Paulo. NBS could be less sensitive in detecting the SV form, which motivated the introduction of a second tier in the Texas NBS program (54). It might have been possible that the female–male imbalance was due to a sampling error and the fact that it was only a 1-year analysis; if we increase the sample size, then this would be corrected. Even if a few cases of the SV form were lost by screening, the reality of patients with the SV form without NBS would be much worse (20).

Our large unscreened cohort comprised patients who were born between 1968 and 2014, all followed in a unique tertiary hospital in Saõ Paulo; therefore, this cohort might be susceptible to bias (12). In Brazil, there is no national database of CAH cases diagnosed in the country, which makes a population-wide study difficult. All patients with CAH in Brazil are referred to tertiary centers, and we know that unscreened cohorts from other CAH centers in São Paulo share similar characteristics with ours,' such as unbalanced female-to-male and SW-to-SV ratios (55–59). The burden in some poorer regions of the country is even worse (60). This is why our cohort seems representative of the entire country. We also stratified the cohort by time and used the most recent data in the sensitivity analyses, which showed partial improvement in the CAH diagnosis; however, the ICER still suggested that NBS is cost-effective.

The main strength of our study was that we were able to assess other benefits of NBS for CAH cases beyond decreasing mortality and use them in CEA analysis, which had never been performed before. Thus far, the main rationale for the economic evaluation of CAH-NBS has been the prevention of deaths due to neonatal SW crisis, and it has been argued that the cost-effectiveness of CAH-NBS should be based on other benefits of early recognition (5). Although some economic evaluations considered hospitalization outcomes in the SW form, long-term outcomes for SV cases have been neglected.

In conclusion, this study emphasizes the importance of NBS in reducing CAH mortality and morbidity, showing that even from an economic point of view, performing CAH-NBS can be beneficial. We believe that our study provides a useful economic assessment to inform decisions on the adoption of CAH screening in NBS programs. However, we encourage researchers in other countries to conduct CEAs on CAH-NBS so that the nation-wide adoption of CAH-NBS can be tailored to the economic situation of the country.

## Data Availability Statement

The raw data supporting the conclusions of this article will be made available by the authors, without undue reservation.

## Ethics Statement

The studies involving human participants were reviewed and approved by Ethics in Research Committees of the Universidade de São Paulo (online register number 14558). Written informed consent to participate in this study was provided by the participants' legal guardian/next of kin.

## Author Contributions

MM: study design, collecting data, and elaborate the manuscript. TB, BM, GM, DD, FC, GH, AC, ET, and LH: study design and manuscript review. All authors contributed to the article and approved the submitted version.

## Conflict of Interest

The authors declare that the research was conducted in the absence of any commercial or financial relationships that could be construed as a potential conflict of interest.

## References

[1] White PC Bachega TA. Congenital adrenal hyperplasia due to 21 hydroxylase deficiency: from birth to adulthood. Semin Reprod Med. (2012) 30:400–9. 10.1055/s-0032-1324724 23044877

[2] Speiser PW White PC. Congenital adrenal hyperplasia. N Engl J Med. (2003) 349:776–88. 10.1056/NEJMra021561 12930931

[3] Hayashi GY Carvalho DF de Miranda MC Faure C Vallejos C Brito VN. Neonatal 17-hydroxyprogesterone levels adjusted according to age at sample collection and birth-weight improve the efficacy of congenital adrenal hyperplasia newborn screening. Clin Endocrinol. (2016) 86:480–7. 10.1111/cen.13292 27978607

[4] Silveira EL dos Santos EP Bachega TA van der Linden Nader I Gross JL Elnecave RH. The actual incidence of congenital adrenal hyperplasia in Brazil may not be as high as inferred–an estimate based on a public neonatal screening program in the state of Goiás. J Pediatr Endocrinol Metab. (2008) 21:455–60. 10.1515/JPEM.2008.21.5.455 18655527

[5] Speiser PW Arlt W Auchus RJ Baskin LS Conway GS Merke DP. Congenital adrenal hyperplasia due to steroid 21-hydroxylase deficiency: an endocrine society clinical practice guideline. J Clin Endocrinol Metab. (2018) 103:4043–88. 10.1210/jc.2018-01865 30272171 PMC6456929

[6] Sarafoglou K Banks K Kyllo J Pittock S Thomas W. Cases of congenital adrenal hyperplasia missed by newborn screening in Minnesota. JAMA. (2012) 307:2371–4. 10.1001/jama.2012.5281 22692165

[7] Votava F Török D Kovács J Möslinger D Baumgartner-Parzer SM Sólyom J. Estimation of the false-negative rate in newborn screening for congenital adrenal hyperplasia. Eur J Endocrinol. (2005) 152:869–74. 10.1530/eje.1.01929 15941926

[8] Kopacek C de Castro SM Prado MJ da Silva CM Beltrão LA Spritzer PM. Neonatal screening for congenital adrenal hyperplasia in Southern Brazil: a population based study with 108,409 infants. BMC Pediatr. (2017) 17:22. 10.1186/s12887-016-0772-x 28095810 PMC5240440

[9] Pezzuti IL Barra CB Mantovani RM Januário JN Silva IN. A three-year follow-up of congenital adrenal hyperplasia newborn screening. J Pediatr. (2014) 90:300–7. 10.1016/j.jped.2013.09.007 24560529

[10] Barra CB Silva IN Pezzuti IL Januário JN. Neonatal screening for congenital adrenal hyperplasia. Rev Assoc Med Bras. (2012). 58:459–64. 10.1590/S0104-42302012000400017 22930025

[11] Hayashi G Faure C Brondi MF Vallejos C Soares D Oliveira E. Weight-adjusted neonatal 17OH-progesterone cutoff levels improve the efficiency of newborn screening for congenital adrenal hyperplasia. Arq Bras Endocrinol Metabol. (2011) 55:632–7. 10.1590/S0004-27302011000800019 22218447

[12] Grosse SD Van Vliet G. How many deaths can be prevented by newborn screening for congenital adrenal hyperplasia? Horm Res. (2007) 67:284–91. 10.1159/000098400 17199092

[13] Coulm B Coste J Tardy V Ecosse E Roussey M Morel Y. Efficiency of neonatal screening for congenital adrenal hyperplasia due to 21-hydroxylase deficiency in children born in mainland France between 1996 and 2003. Arch Pediatr Adolesc Med. (2012) 166:113–20. 10.1001/archpediatrics.2011.774 22312171

[14] Brosnan CA Brosnan P Therrell BL Slater CH Swint JM Annegers JF. A comparative cost analysis of newborn screening for classic congenital adrenal hyperplasia in Texas. Public Health Rep. (1998) 113:170–8. 9719819 PMC1308657

[15] Heather NL Seneviratne SN Webster D Derraik JG Jefferies C Carll J. Newborn screening for congenital adrenal hyperplasia in New Zealand, 1994-2013. J Clin Endocrinol Metab. (2015) 100:1002–8. 10.1210/jc.2014-3168 25494862

[16] Gidlöf S Wedell A Guthenberg C von Döbeln U Nordenström A. Nationwide neonatal screening for congenital adrenal hyperplasia in sweden: a 26-year longitudinal prospective population-based study. JAMA Pediatr. (2014) 168:567–74. 10.1001/jamapediatrics.2013.5321 24733564

[17] Yoo BK Grosse SD. The cost effectiveness of screening newborns for congenital adrenal hyperplasia. Public Health Genomics. (2009) 12:67–72. 10.1159/000156115 19039250 PMC6706242

[18] Carroll AE Downs SM. Comprehensive cost-utility analysis of newborn screening strategies. Pediatrics. (2006) 117 (5 Pt. 2):S287–95. 10.1542/peds.2005-2633H 16735255

[19] Fox DA Ronsley R Khowaja AR Haim A Vallance H Sinclair G. Clinical impact and cost efficacy of newborn screening for congenital adrenal hyperplasia. J Pediatr. (2020) 220:101–8.e2. 10.1016/j.jpeds.2019.12.057 32044100

[20] de Miranda MC Haddad LBdP Madureira G Mendonca BBd Bachega TASS. Adverse outcomes and economic burden of congenital adrenal hyperplasia late diagnosis in the newborn screening absence. J Endocrine Society. (2019) 4:bvz013. 10.1210/jendso/bvz013 32047870 PMC7003980

[21] Van der Kamp HJ Wit JM. Neonatal screening for congenital adrenal hyperplasia. Eur J Endocrinol. (2004) 151 (Suppl. 3):U71–5. 10.1530/eje.0.151u071 15554889

[22] Gidlöf S Falhammar H Thilén A von Döbeln U Ritzén M Wedell A. One hundred years of congenital adrenal hyperplasia in Sweden: a retrospective, population-based cohort study. Lancet Diabetes Endocrinol. (2013) 1:35–42. 10.1016/S2213-8587(13)70007-X 24622265

[23] Brunelli VL Russo G Bertelloni S Gargantini L Balducci R Chiesa L. Final height in congenital adrenal hyperplasia due to 21-hydroxylase deficiency: the Italian experience. J Pediatr Endocrinol Metab. (2003) 16 (Suppl. 2):277–83. 12729404

[24] Van der Kamp HJ Otten BJ Buitenweg N De Muinck Keizer-Schrama SM Oostdijk W Jansen M. Longitudinal analysis of growth and puberty in 21-hydroxylase deficiency patients. Arch Dis Child. (2002) 87:139–44. 10.1136/adc.87.2.139 12138066 PMC1719187

[25] Donaldson MD Thomas PH Love JG Murray GD McNinch AW Savage DC. Presentation, acute illness, and learning difficulties in salt wasting 21-hydroxylase deficiency. Arch Dis Child. (1994) 70:214–8. 10.1136/adc.70.3.214 8135566 PMC1029745

[26] Husereau D Drummond M Petrou S Carswell C Moher D Greenberg D. Consolidated health economic evaluation reporting standards (CHEERS)–explanation and elaboration: a report of the ISPOR health economic evaluation publication guidelines good reporting practices task force. Value Health. (2013) 16:231–50. 10.1016/j.jval.2013.02.002 23538175

[27] SIGTAP. Sistema de Gerenciamento da Tabela de Procedimentos, Medicamentos, Órteses e Próteses do SUS. Available online at: http://sigtap.datasus.gov.br/tabela-unificada/app/sec/inicio.jsp (accessed July, 2018).

[28] Gov.BR. Banco Central do Brasil. Gov.BR. Available online at: http://www.bcb.gov.br/pt-br/#!/home (accessed July, 2018).

[29] Quintos JB Vogiatzi MG Harbison MD New MI. Growth hormone therapy alone or in combination with gonadotropin-releasing hormone analog therapy to improve the height deficit in children with congenital adrenal hyperplasia. J Clin Endocrinol Metab. (2001) 86:1511–7. 10.1210/jc.86.4.1511 11297576

[30] Instituto Brasileiro de Geografia e Estatística. Tábua Completa de Mortalidade Para o Brasil. (2017). Available online at: https://www.ibge.gov.br/estatisticas/sociais/populacao/9126-tabuas-completas-de-mortalidade.html?edicao=23111&t=sobre (accessed July, 2018).

[31] Ministério da Saúde. DATASUS. Sistema de Informação Sobre Nascidos Vivos - SINASC. Available online at: http://tabnet.datasus.gov.br/cgi/tabcgi.exe?sinasc/cnv/nvuf.def (accessed July, 2018).

[32] Wilson JM Jungner YG. [Principles and practice of mass screening for disease]. Bol Oficina Sanit Panam. (1968) 65:281–393. 4234760

[33] Grosse SD Thompson JD Ding Y Glass M. The use of economic evaluation to inform newborn screening policy decisions: the Washington state experience. Milbank Q. (2016) 94:366–91. 10.1111/1468-0009.12196 27265561 PMC4911729

[34] Van der Kamp HJ Noordam K Elvers B Van Baarle M Otten BJ Verkerk PH. Newborn screening for congenital adrenal hyperplasia in the Netherlands. Pediatrics. (2001) 108:1320–4. 10.1542/peds.108.6.1320 11731654

[35] Brosnan PG Brosnan CA Kemp SF Domek DB Jelley DH Blackett PR. Effect of newborn screening for congenital adrenal hyperplasia. Arch Pediatr Adolesc Med. (1999) 153:1272–8. 10.1001/archpedi.153.12.1272 10591305

[36] Steigert M Schoenle EJ Biason-Lauber A Torresani T. High reliability of neonatal screening for congenital adrenal hyperplasia in Switzerland. J Clin Endocrinol Metab. (2002) 87:4106–10. 10.1210/jc.2002-012093 12213856

[37] Edelman S Desai H Pigg T Yusuf C Ojodu J. Landscape of Congenital Adrenal Hyperplasia Newborn Screening in the United States. Int J Neonatal Screen. (2020) 6:64. 10.3390/ijns6030064 33239590 PMC7569894

[38] Cheng TQ Speiser PW. Treatment outcomes in congenital adrenal hyperplasia. Adv Pediatr. (2012) 59:269–81. 10.1016/j.yapd.2012.04.009 22789582

[39] Knowles RL Khalid JM Oerton JM Hindmarsh PC Kelnar CJ Dezateux C. Late clinical presentation of congenital adrenal hyperplasia in older children: findings from national paediatric surveillance. Arch Dis Child. (2014) 99:30–4. 10.1136/archdischild-2012-303070 24043550 PMC3888619

[40] Neumann PJ Cohen JT Weinstein MC. Updating cost-effectiveness–the curious resilience of the $50,000-per-QALY threshold. N Engl J Med. (2014) 371:796–7. 10.1056/NEJMp1405158 25162885

[41] Marseille E Larson B Kazi DS Kahn JG Rosen S. Thresholds for the cost-effectiveness of interventions: alternative approaches. Bull World Health Organ. (2015) 93:118–24. 10.2471/BLT.14.138206 25883405 PMC4339959

[42] Instituto Brasileiro de Geografia e Estatística. Brasil em Síntese. Contas Nacionais. PIB Per Capta. IBGE. Available online at: http://brasilemsintese.ibge.gov.br/contas-nacionais/pib-per-capita.html (accessed July, 2018).

[43] Hird BE Tetlow L Tobi S Patel L Clayton PE. No evidence of an increase in early infant mortality from congenital adrenal hyperplasia in the absence of screening. Arch Dis Child. (2014) 99:158–64. 10.1136/archdischild-2013-304473 24225272

[44] Wu JY Sudeep Cowley DM Harris M McGown IN Cotterill AM. Is it time to commence newborn screening for congenital adrenal hyperplasia in Australia? Med J Aust. (2011) 195:260–2. 10.5694/mja11.10284 21895585

[45] Gong LF Gao X Yang N Zhao JQ Yang HH Kong YY. A pilot study on newborn screening for congenital adrenal hyperplasia in Beijing. J Pediatr Endocrinol Metab. (2019) 32:253–8. 10.1515/jpem-2018-0342 30817302

[46] Kovács J Votava F Heinze G Sólyom J Lebl J Pribilincová Z. Lessons from 30 years of clinical diagnosis and treatment of congenital adrenal hyperplasia in five middle European countries. J Clin Endocrinol Metab. (2001) 86:2958–64. 10.1210/jc.86.7.2958 11443151

[47] Shetty VB Bower C Jones TW Lewis BD Davis EA. Ethnic and gender differences in rates of congenital adrenal hyperplasia in Western Australia over a 21 year period. J Paediatr Child Health. (2012) 48:1029–32. 10.1111/j.1440-1754.2012.02584.x 23039988

[48] Nordenström A Ahmed S Jones J Coleman M Price DA Clayton PE. Female preponderance in congenital adrenal hyperplasia due to CYP21 deficiency in England: implications for neonatal screening. Horm Res. (2005) 63:22–8. 10.1159/000082896 15627780

[49] Instituto Brasileiro de Geografia e Estatística. Tabuas de Mortalidades Brasil. IBGE (2017). Available online at: https://www.ibge.gov.br/estatisticas/sociais/populacao/9126-tabuas-completas-de-mortalidade.html?edicao=23111&t=resultados (accessed July, 2018).

[50] Nass R Baker S. Learning disabilities in children with congenital adrenal hyperplasia. J Child Neurol. (1991) 6:306–12. 10.1177/088307389100600404 1940131

[51] Winfeld M Patel P Shah B Nass R Milla S. Early occurrence of cerebral white matter abnormality detected in a neonate with salt-wasting congenital adrenal hyperplasia. J Pediatr Endocrinol Metab. (2013) 26:13–7. 10.1515/jpem-2012-0154 23382298

[52] Van der Linde AAA Schönbeck Y van der Kamp HJ van den Akker ELT van Albada ME Boelen A. Evaluation of the Dutch neonatal screening for congenital adrenal hyperplasia. Arch Dis Child. (2019) 104:653–7. 10.1136/archdischild-2018-315972 30712004

[53] Pode-Shakked N Blau A Pode-Shakked B Tiosano D Weintrob N Eyal O. Combined gestational age- and birth weight-adjusted cutoffs for newborn screening of congenital adrenal hyperplasia. J Clin Endocrinol Metab. (2019) 104:3172–80. 10.1210/jc.2018-02468 30865229

[54] Therrell BL Berenbaum SA Manter-Kapanke V Simmank J Korman K Prentice L. Results of screening 1.9 million Texas newborns for 21-hydroxylase-deficient congenital adrenal hyperplasia. Pediatrics. (1998) 101 (4 Pt. 1):583–90. 10.1542/peds.101.4.583 9521938

[55] Paulino LC Araujo M Guerra G Marini SH De Mello MP. Mutation distribution and CYP21/C4 locus variability in Brazilian families with the classical form of the 21-hydroxylase deficiency. Acta Paediatr. (1999) 88:275–83. 10.1111/j.1651-2227.1999.tb01096.x 10229037

[56] Torres N Mello MP Germano CM Elias LL Moreira AC Castro M. Phenotype and genotype correlation of the microconversion from the CYP21A1P to the CYP21A2 gene in congenital adrenal hyperplasia. Braz J Med Biol Res. (2003) 36:1311–8. 10.1590/S0100-879X2003001000006 14502362

[57] Coeli-Lacchini FB Turatti W Elias PC Elias LL Martinelli CE Moreira AC. A rational, non-radioactive strategy for the molecular diagnosis of congenital adrenal hyperplasia due to 21-hydroxylase deficiency. Gene. (2013) 526:239–45. 10.1016/j.gene.2013.03.082 23570880

[58] Caldato MC Fernandes VT Kater CE. One-year clinical evaluation of single morning dose prednisolone therapy for 21-hydroxylase deficiency. Arq Bras Endocrinol Metabol. (2004) 48:705–12. 10.1590/S0004-27302004000500017 15761542

[59] Guerra-Junior G Grumach AS de Lemos-Marini SH Kirschfink M Condino Neto A de Araujo M. Complement 4 phenotypes and genotypes in Brazilian patients with classical 21-hydroxylase deficiency. Clin Exp Immunol. (2009) 155:182–8. 10.1111/j.1365-2249.2008.03838.x 19137635 PMC2675248

[60] Campos VC Pereira RM Torres N Castro M Aguiar-Oliveira MH. High frequency of Q318X mutation in patients with congenital adrenal hyperplasia due to 21-hydroxylase deficiency in northeast Brazil. Arq Bras Endocrinol Metabol. (2009) 53:40–6. 10.1590/S0004-27302009000100007 19347184

